# Incidence, risk factors and outcomes of seizures occurring after craniotomy for primary brain tumor resection

**DOI:** 10.17712/nsj.2017.2.20160570

**Published:** 2017-04

**Authors:** Hasan M. Al-Dorzi, Abdullah A. Alruwaita, Bothaina O. Marae, Bushra S. Alraddadi, Hani M. Tamim, Ahmad Ferayan, Yaseen M. Arabi

**Affiliations:** *From the Intensive Care Department (Al-Dorzi, Tamim, Arabi), from the Department of Surgery/Neurosurgery (Ferayan), King Abdullah International Medical Research Center and King Saud bin Abdulaziz University for Health Sciences, Riyadh, from the College of Medicine (Alruwaita), King Saud University, Riyadh, from the Department of Surgery (Marae), Taibah University, and Ministry of Health (Alraddadi), Al-Madinah Al-Munawarah, Kingdom of Saudi Arabia*

## Abstract

**Objective:**

To determine the incidence, risk factors and outcomes of early post-craniotomy seizures.

**Method:**

This was a retrospective cohort study of all patients who underwent craniotomy for primary brain tumor resection (2002-2011) and admitted postoperatively to the intensive care unit. The patients were divided into 2 groups depending on the occurrence of seizures within 7 days.

**Results:**

One-hundred-ninety-three patients were studied: 35.8% had preoperative seizure history and 16.6% were on prophylactic antiepileptic drugs (AEDs). Twenty-seven (14%) patients had post-craniotomy seizures. The tumors were mostly meningiomas (63% for the post-craniotomy seizures group versus 58.1% for the other group; *p*=0.63) and supratentorial (92.6% for the post-craniotomy seizures versus 78.4% for the other group, *p*=0.09) with tumor diameter=3.7±1.5 versus 4.2±1.6 cm, (*p*=0.07). One (3.1%) of the 32 patients on prophylactic AEDs had post-craniotomy seizures compared with 12% of the 92 patients not receiving AEDs preoperatively (*p*=0.18). On multivariate analysis, predictors of post-craniotomy seizures were preoperative seizures (odds ratio, 2.62; 95% confidence interval, 1.12-6.15) and smaller tumor size <4 cm (odds ratio, 2.50; 95% confidence interval, 1.02-6.25). Post-craniotomy seizures were not associated with increased morbidity or mortality.

**Conclusion:**

Early seizures were common after craniotomy for primary brain tumor resection, but were not associated with worse outcomes. Preoperative seizures and smaller tumor size were independent risk factors.

Primary brain tumors are relatively rare. According to the Central Brain Tumor Registry of the United States, the overall incidence was estimated at 18.1 per 100000 person-years and the 2010 prevalence was 221.8 per 100000 people.^1^ Most (66.2%) tumors are either gliomas or meningiomas, which are approximately equally distributed.^1^ Seizures are commonly associated with primary brain tumors with 20-40% of all brain tumor patients have had a seizure at diagnosis.^2^ Seizure incidence varies depending on the tumor histology and location.^3^ Incidence is higher in tumors affecting the temporal, parietal and frontal lobes than other lobes and in cortical than deeper tumors.^3^ Older age and tumor size are inversely associated with seizures.^4^ Surgical resection through craniotomy is the main treatment of primary brain tumors. Post-craniotomy seizures are relatively common, but studies have shown a wide range of incidence. In a study published in 1985, the incidence of post-operative seizures in 68 glioma patients who had no previous seizures was 29%.^5^ Another study found that 23.6% of 72 consecutive patients who underwent craniotomy and palliative resection for glioblastoma multiforme had postoperative seizures.^6^ More recently, in a study of 180 patients with no preoperative seizures who underwent resection of a convexity meningioma, seizures occurred in only 2 (1.1%) patients.^7^ Another recent study found a seizure incidence of 8% in 123 patients undergoing craniotomy for metastatic brain tumors or gliomas.^8^ Of note, these studies differed in their patient populations which may have affected results. The use of antiepileptic drugs (AEDs) in the perioperative period for primary seizure prevention is controversial.^9^ The American Academy of Neurology recommends against AED prophylaxis in the management of brain tumors.^2^ A systematic review 39 observational case series found similar rate of new post-craniotomy seizures with or without perioperative prophylactic AED in supratentorial meningioma patients.^10^ Another systematic review of 19 studies involving 698 patients on the role of AEDs in the perioperative care of patients with supratentorial meningioma resection found no significant differences in perioperative mortality between the AED and no-AED cohorts.^11^ However, AED therapy is commonly used for primary seizure prevention. A survey of neurosurgeons to evaluate practice patterns, more than 70% of respondents reported routine use of AED prophylaxis for patients with intra-axial gliomas or brain metastases.^12^ On multivariate analysis, the number of years in practice of American Board of Neurosurgery-certified surgeons was the strongest predictor for the use of AED prophylaxis.^12^

Seizures occurring after craniotomy are considered an added burden as they can be complicated by injury, aspiration, respiratory failure and even death. Recent studies on their incidence, risk factors and outcomes are relatively scarce. The objectives of this study were to determine the incidence of early seizures after craniotomy for primary brain tumor resection, study their risk factors including AED therapy and study their outcomes.

## Methods

### Patients and setting

This study was a retrospective cohort of all patients admitted to the intensive care unit (ICU) of King Abdulaziz Medical City in Riyadh, Saudi Arabia between 01/01/2002 and 31/12/2011 after craniotomy for total or partial resection of a primary brain tumor. The ICU admitted medical and surgical patients and worked as a closed unit in which onsite board-certified intensivists were directly responsible for the provision of intensive care management on a 24 hours a day, 7 days a week basis.^13^ The intensivists worked collaboratively with a multidisciplinary team of critical care fellows and registrars, critical care nurses, respiratory therapists, clinical pharmacists, nutritionists, physical therapists and others. All patients who underwent craniotomy for brain tumor resection were routinely admitted to the ICU postoperatively. The post-operative care was primarily by the ICU team. The neurosurgery team consisted of a neurosurgery consultant and a registrar and followed patients postoperatively on a daily and as needed basis. The Institutional Review Board of King Saud bin Abdulaziz University for Health Sciences approved this study and granted waiver of consent.

### Collected data

The patients were divided into 2 groups depending on the occurrence of early post-craniotomy seizures defined as any clinical seizure occurring within 7 days after craniotomy. The following additional information were noted: baseline characteristics, admission Acute Physiology and Chronic Health Evaluation (APACHE) II score,^14^ which assesses the severity of illness in ICU patients and allows estimation of in-hospital mortality, the average diameter of the tumor based on preoperative head computed tomography, tumor location and histology, previous seizure history and characteristics, AED use before and after craniotomy, extent of surgery and its duration. Studied outcomes were requirement for reintubation within 7 days of ICU admission, duration of mechanical ventilation, development of hospital-acquired pneumonia, occurrence of Steven Johnson syndrome as a possible side effect of AED, development of venous thromboembolism, length of stay in the ICU and hospital and ICU and hospital mortality.

### Statistical analysis

Statistical analysis was performed using Statistical Analysis System (SAS, version 9.0; SAS Institute, Cary, NC). Continuous variables were presented as means with standard deviations and categorical variables as frequencies with percentages. The Chi-square or Fisher’s exact test was used to assess differences between categorical variables. The Student’s t-test and, when indicated, the Mann-Whitney U test were performed to assess differences between continuous variables. To assess the risk factors of post-craniotomy seizures, multivariate analysis was performed with the following variables entered in the model: age, gender, body mass index, preoperative antiepileptic drug therapy (primary and secondary prevention with no therapy being the reference), tumor size (categorized based on the median tumor diameter as ≥ 4 cm versus < 4 cm), tumor location (frontal versus other lobe involvement and anterior versus posterior fossa location) and tumor histology (meningioma versus other tumor histology). The results were presented as odds ratios (ORs) with 95% confidence intervals (CIs).

## Results

### Patient characteristics

In the 10-year period, 193 patients had craniotomy for primary brain tumor resection and were admitted to the ICU. The characteristics of these patients are found in **Table 1**. They had a mean age of 45.7±15.4 years and were predominantly (64.8%) males. Almost half (47.8%) of the patients were obese (body mass index ≥ 30 kg/m^2^). Most of the tumors were meningiomas (58.5%) and supratentorial (80.3%). Sixty-nine (35.8%) patients had seizure history before craniotomy. Preoperative seizures were generalized in most patients (57.1%) and partial in 38.9% of them. Only 3.7% of patients had status epilepticus. Thirty-two (16.5%) patients were on AED prophylaxis (25 patients on phenytoin, 3 on carbamazepine and 4 on other AEDs).

**Table 1:** Characteristics of patients according to the incidence of seizures within 7 days after craniotomy for primary brain tumor resection.

Variables	All patients N=193	Post-craniotomy sz N=27	No postoperative sz N=166	*P*-value
Age (years), mean±SD	45.7±15.4	44.7±16.7	46.0±15.2	0.69**
Male Gender, N (%)	125 (64.8)	17 (63.0)	108 (65.1)	0.83
BMI (Kg/m^2^), mean±SD	30.2±7.2	32.8±7.3	29.7±7.2	0.053**
APACHE II score, mean±SD	12.8±5.9	12.9±4.1	12.8±6.3	0.12**
*Comorbid conditions*				
N(%)Hypertension	55 (28.5)	10 (37.0)	45 (27.1)	0.29
Diabetes	55 (28.5)	6 (22.2)	49 (29.5)	0.44
Preoperative seizures*	69 (35.8)	15 (55.6)	54 (32.5)	0.04
N(%)Generalized	31 (57.1)	8 (57.1)	23 (57.5)	0.68
Focal Status	21 (38.9)	6 (42.9)	15 (37.5)	
epilepticus	2 (3.7)	0 (0)	2 (5.0)		
*Preoperative antiepileptic drugs N(%)*	101(52.3)	16 (59.3)	85 (51.2)	0.57
Primary prevention	32 (16.6)	1 (3.7)	31 (18.7)	0.054
Secondary prevention	69 (35.8)	15 (55.6)	54 (32.5)	0.04
No antiepileptic drugs	92 (47.7)	11 (40.7)	81 (48.8)	0.57
*Tumor histology N(%)*				0.33
Low-grade glioma	23 (11.9)	3 (11.1)	20 (12.0)	
High-grade glioma	33 (17.1)	4 (14.8)	29 (17.5)	
Meningioma	113 (58.5)	17 (63.0)	96 (57.8)	
Others	24 (12.4)	3 (11.1)	21 (12.6)
*Tumor location N(%)*				
Frontal involvement	53 (27.5)	9 (33.3)	44 (26.5)	0.61
Parietal involvement	44 (22.8)	5 (18.5)	39 (23.4)	0.75
Temporal involvement	27 (14.0)	4 (14.8)	23 (13.9)	1.0
Skull base	32 (16.6)	3 (11.1)	29 (17.5)	0.58
Posterior fossa	38 (19.7)	2 (7.4)	36 (21.7)	0.12
*Tumor site N(%)*				0.40
Right	76 (39.4)	12 (44.4)	64 (38.6)	
Left	86 (44.6)	9 (33.3)	77 (46.4)	
Central	31 (16.1)	6 (22.2)	25 (15.1)	
Tumor size/ diameter (cm)	4.1±1.6	3.5±1.5	4.2±1.6	0.07**
*Characteristics of surgery N(%)*				
Subtotal resection	54 (28.0)	6 (22.2)	48 (28.9)	0.46
Re-do craniotomy	40 (20.7)	5 (18.5)	35 (21.1)	0.76
Surgery duration (hrs), mean ± SD	4.69±2.52	4.41±1.72	4.74±2.63	0.53^†^
*Laboratory data on the day ICU admission/ seizure, mean±SD*				
Blood glucose (mmol/L)	9.2±3.5	9.3±4.0	9.1±3.4	0.86
BUN (mmol/L)	4.4±2.2	4.9±2.9	4.3±2.1	0.29
Creatinine (µmol/L)	66.6±17.4	68.0±17.2	66.4±17.6	0.70
Sodium (mmol/L)	138±4	138±3	138±4	0.41
Calcium (mmol/L)	2.00±0.29	1.98±0.29	2.01±0.29	0.66
Magnesium (mmol/L)	0.75±0.14	0.74±0.12	0.75±0.15	0.71
Phosphorous (mmol/L)	1.30±0.35	1.30±0.38	1.30±0.35	0.95
Phenytoin (µmoI/L)	46.0±28.2	45.1±24.0	46.3±29.6	0.90*

AED - antiepileptic drug, APACHE - Acute Physiology and Chronic Health Evaluation, BMI - body mass index, BUN - Blood urea nitrogen, ICU - intensive care unit, Sz - seizure,

* seizure characteristics available for 54 patients,

** equality of variance > 0.05,

† equality of variance < 0.05,*p*-value of the Mann-Whitney Test (non-parametric)=0.08

### Incidence and risk factors for post-craniotomy seizures

Twenty-seven (14.0%) patients had post-craniotomy clinical seizures. **Table 1** describes the characteristics of patients who had and did not have post-craniotomy seizures. Twelve patients (44.4%) had seizures within the first 24 hours after craniotomy. The duration of seizures ranged between 0.5 and 7 minutes; 19 events were described as partial and 4 as generalized tonic clonic. The serum electrolyte profile was not different between patients with and without post-operative seizures. Phenytoin was used for seizure control in 25 (92.6%) patients and its level was not different from that of patients on phenytoin but did not have post-craniotomy seizures (45±45 and 46±30 µmol/L; *p*=0.90). Phenytoin level was subtherapeutic in 30.7% of post-craniotomy patients and 40.5% of the other patients (*p*=0.74). Of note, the phenytoin level was similar in obese and nonobese patients (42±25 and 47±35 µmol/L, respectively; *p*=0.72).

Compared with all other patients, patients who developed post-craniotomy seizures had similar age, gender, APACHE II score, surgery duration (4.4±1.7 versus 4.7±2.6 hours, *p*=0.41) and frontal lobe involvement (33.3% versus 26.5%, *p*=0.61), but more (55.6%) of them had previous seizures (versus 32.9%, *p*=0.02). Patients who had post-craniotomy seizures tended to have higher body mass index than those who did not (32.8±7.3 versus 29.7±7.2 kg/m^2^, *p*=0.053). The incidence of post-craniotomy seizures was significantly higher in the obese patients (20.8%) than the nonobese (0.5%; *p*=0.04). Most tumors were meningiomas in both groups (63.0% versus 57.8%, *p*=0.77). The tumors tended to be smaller (3.5±1.5 versus 4.2±1.6 cm, *p*=0.07) and more supratentorial (92.6% versus 78.3%, *p*=0.08) in post-craniotomy seizure patients than all other patients.

The incidence of post-craniotomy seizures was 21.7% in patients with previous history of seizures, 12.0% in those without such history and not taking AED and 3.1% in those taking AED for primary seizure prevention (*p*=0.03 for the difference between the three groups). The type of preoperative seizures was not associated with post-craniotomy seizure incidence (**Table 1**). However, the two patients with preoperative status epilepticus had seizures after craniotomy. Its incidence was 12.5% in gliomas and 15.0% in meningiomas (*p*=0.83); and was 15.8% in tumors located in the right fossa compared with 10.5% for tumors in the left fossa (*p*=0.44).

On multivariate analysis, the following variables were independent predictors of post-craniotomy seizures: preoperative seizures (OR, 2.62; 95% CI, 1.12-6.15 with patients without seizures and not on AED prophylaxis being the reference group) and tumor size < 4 cm (OR, 2.50; 95% CI, 1.02-6.25 versus larger tumors). Antiepileptic drugs prophylaxis was not associated with post-craniotomy seizures.

### Outcomes of patients with post-craniotomy seizures

**Table 2** describes the outcomes of patients. Compared with those who did not have post-craniotomy seizures, patients with early post-craniotomy seizures had similar outcomes: hospital mortality (14.8% versus 9.0%, *p*=0.31), length of stay in the ICU (2.9±4.0 versus 4.0±5.7 days, *p*=0.27) and hospital (40.1±48.0 versus 44.8±57.8 days, *p*=0.68), tracheostomy (11.1% versus 10.2%, *p*=1.0), hospital acquired pneumonia (0% versus 1.8%, *p*=1.0) and venous thromboembolism (3.7% versus 5.4%, *p*=1.0).

**Table 2:** Outcomes of patients according to the incidence of seizures within 7 days after craniotomy for primary brain tumor resection.

Outcomes	All patients N=193	Post-craniotomy sz N=27	No postoperative sz N=166	*P*-value
Hospital mortality N(%)	19 (9.8)	4 (14.8)	15 (9.0)	0.31
ICU mortality N(%)	4 (2.1)	1 (3.7)	3 (1.8)	0.46
ICU LOS (days), mean±SD	4.0±5.6	2.9±4.0	4.0±5.8	0.27**
Hospital LOS (days), mean±SD	44.2±56.4	40.1±48.1	44.8±57.8	0.68*
Duration of MV (days), mean±SD	1.6±3.3	1.7±3.9	1.6±3.2	0.82*
Reintubation within 7 days N(%)	10 (5.2)	1 (3.7)	9 (5.4)	1.0
Hospital-acquired pneumonia N(%)	3 (1.6)	0 (0)	3 (1.8)	1.0
Tracheostomy N(%)	20 (10.4)	3 (11.1)	17 (10.2)	1.0
Venous thromboembolism N(%)	10 (5.2)	1 (3.7)	9 (5.4)	1.0
Pulmonary embolism N(%)	5 (2.6)	0 (0)	5 (3.0)	1.0
Deep venous thrombosis N(%)	7 (3.6)	1 (3.7)	6 (3.6)	1.0
Steven Johnson syndrome N(%)	1 (0.5)	0 (0)	1 (0.6)	1.0

ICU - intensive care unit, LOS - length of stay, MV - mechanical ventilation, Sz - seizure,

* equality of variance>0.05,

** equality of variance <0.05,*p*-value of the Mann-Whitney Test (non-parametric)=0.27

## Discussion

The main findings of this study were the following: early post-craniotomy seizures were relatively common occurring in 14% of patients; their risk factors were previous history of seizures and smaller tumor size (average diameter <4 cm); prophylactic AED did not reduce their incidence; and they were not associated with increased morbidity or mortality.

Craniotomy with surgical resection is the main treatment of primary brain tumors. Post-craniotomy seizures have been reported to occur at different rates that ranged from 1.1 to 29%^5^-^7^,^15^,^16^ possibly because of the different patient case-mix of the studies. Boarini et al^5^ reported the incidence (29%) in patients with gliomas. Sugrue et al^7^ studied the incidence (1.1%) in patients who had convexity meningioma with no preoperative seizures. Wu et al^8^ assessed the incidence (8%) in patients with metastatic brain tumors or gliomas. In our study the incidence of seizures was 14%. Several mechanisms may be proposed to explain post-craniotomy seizures and include acute tissue damage, tissue hypoxia and edema.^16^ These surgery-related effects, among other factors,^9^ may induce epileptogenic activity. In our study, post-craniotomy seizures were more common in the obese than nonobese patients. The relation between obesity and seizure is not clear. It is suggested that obesity is common in patients with epilepsy due to the effect of epilepsy on physical activity and AEDs on central and peripheral mechanisms regulating weight homeostasis including leptin and insulin.^17^ Obesity-induced sub-therapeutic AED level is another consideration. Independent predictors of post-craniotomy seizures were previous seizure history and smaller tumor size (average diameter <4 cm). Preoperative seizure history is well known to increase post-craniotomy seizures.^16^ The relationship between tumor size and post-craniotomy seizures is unclear. It is postulated that rapidly growing tumors, especially those situated in deeper structures, present with symptoms other than seizures. Lee et al^15^ found that larger tumor size was associated with presentation with seizures (OR, 1.31 per cm^3^; 95% CI, 1.06-1.60) in low-grade but not high-grade tumors. Similar to our findings, Telfeian et al^18^ observed that smaller glioblastoma multiforme tumor size was associated increased risk of postoperative seizue. More dissection of brain tissue may be needed to reach smaller tumors and so may explain the higher risk of post-craniotomy seizures for smaller tumors. Other characteristics that increase seizure risk, such tumor depth from surface, may have been present but not studied.

The use of AEDs in seizure treatment and for secondary prevention is the standard of care.^2^,^19^ However, their use for primary prevention in patients with brain tumors is debatable. A metaanalysis of 5 trials that evaluated AED prophylaxis in patients with brain tumors (primary glial tumors, cerebral metastases, and meningiomas) found that the 3 studied AEDs (phenobarbital, phenytoin, and valproic acid) had no effect on seizure prevention at one week (OR, 0.91; 95% [CI, 0.45-1.83) and at 6 months (OR, 1.01; 95% CI, 0.51-1.98).^20^ This effect was observed for primary glial tumors (OR, 3.46; 95% CI, 0.32-37.47), cerebral metastases (OR, 2.50; 95% CI, 0.25-24.72) and meningiomas (OR, 0.62; 95% CI, 0.10-3.85).^20^ The ineffectiveness of AED prophylaxis was additionally associated with increased risk of an adverse event (relative risk, 6.10; 95% CI, 1.10-34.63; *p*=0.046) compared with no AED.^21^ Despite this, the use of AED for to prevent seizures after craniotomy is common.^12^,^22^ In our study 32 out of 124 (25.8%) patients without preoperative seizures received AED, mostly phenytoin, for seizure prevention. The evidence for such practice is evolving. A systematic review of 6 randomized trials involving 1398 patients who underwent craniotomy for different pathologies found little evidence to suggest that prophylactic AEDs is effective in preventing post-craniotomy seizures with the available trials differing in methodologies and being inconsistent in outcomes reporting.^23^ Phenytoin seems to be ineffective in preventing post-craniotomy seizures. A recent randomized controlled trial was stopped early as the incidence of seizures within 30 days of craniotomy for resection of metastatic brain tumors or gliomas was not different in the phenytoin group compared with controls (8% and 10%, respectively; *p*=1.0).^8^ It is believed that an AED with no major drug-drug interaction and more favorable adverse event profile, such as levetiracetam, would be a more appropriate choice for trials on post-craniotomy seizure prophylaxis.^24^ An observational study showed that seizure incidence in the immediate post-craniotomy period was only 7.3% in patients with brain tumors at higher risk for postoperative seizures who were postoperatively treated with levetiracetam.^25^ A phase II trial compared levetiracetam with phenytoin and found lower incidence of early post-craniotomy seizures 1.4% versus 15.1% *p*=0.005 and lower rate of side effects in the levetiracetam group.^26^

The patients who developed post-craniotomy seizures had benign hospital course compared with other patients. Seizure after craniotomy is a known risk factor for tracheal reintubation.^27^ In our study, the patients who developed seizure did not need reintubation within 7 days of craniotomy more than other patients neither they stayed in the ICU for longer period. Other outcomes were similar including ICU and hospital mortality, requirement for tracheostomy and occurrence of venous thromboembolism.

The findings of this study should be interpreted in the light of its strengths and limitations. The study was conducted at a single center. The retrospective nature of this study does not allow controlling or accounting for a number of factors. These factors include preoperative seizure characteristics, seizure management, tumor depth and surgery details. Most post-craniotomy seizures were partial and only 4 patients had generalized seizures. The differences in outcomes may have been statistically insignificant because of the small sample size. On the other hand, this study includes a relatively high number of patients with primary brain tumors of different histology undergoing surgical resection and reflects general neurosurgery practice.

In conclusion, early seizures were common after craniotomy for primary brain tumor resection, but were not associated with worse outcomes. Prior seizure history and smaller tumor size were independent risk factors for post-craniotomy seizures. Additionally, preoperative AEDs for primary seizure prevention were not associated with lower incidence of seizures after craniotomy and so this practice may not be warranted which is commensurate with the current recommendations.
